# Who is she? Changes in the person context affect categorization

**DOI:** 10.3389/fpsyg.2013.00745

**Published:** 2013-10-14

**Authors:** Elizabeth R. Goldenberg, Catherine M. Sandhofer

**Affiliations:** Department of Psychology, University of CaliforniaLos Angeles, CA, USA

**Keywords:** context, generalization, categorization, word learning, cognitive development, memory

## Abstract

Changes between the learning and testing contexts affect learning, memory, and generalization. We examined whether a change (between learning and testing) in the person children were interacting with affects generalization. Three-, four-, and five-year-old children were trained on eight novel noun categories by one experimenter. Children were tested for their ability to generalize the label to a new category member by either the same experimenter who trained them or by a novel experimenter. Three-year-old children's performance was not affected by who they were tested by. Four- and five-year-old children's performance was lower when tested by the novel experimenter. The results are discussed in terms of source monitoring and the effect of perceptual context change on category generalization.

Context experienced during learning has been broadly and robustly shown to affect recall and generalization (e.g., Godden and Baddeley, 1975; Smith, 1982; Amabile and Rovee-Collier, 1991; Hayne et al., 2000; Learmonth et al., 2004; Robinson and Pascalis, 2004). For example, in Godden and Baddeley's (1975) seminal study, adults studied a list of words either on land or under water and were later asked to recall the list in one of the two contexts. Memory was lower when the learning and recall contexts did not match (e.g., tested on land when training took place under water) than when learning and recall contexts did match (e.g., tested on land when training took place on land). Based on these and other results, learning is considered to be “context dependent.” That is, retrieval is highly dependent on the state in which the information was learned. Differences in cues between learning and retrieval reduce memory performance. One strong contextual factor for children is the people they learn from. Human interaction is omnipresent in young children's lives (Tomasello, 1992; Akhtar and Tomasello, 2000; Meltzoff et al., 2012). The goal of this study is to examine how the person a child interacts with affects context dependent learning across development.

Context effects have been demonstrated across multiple types of contexts and across a wide range of paradigms, species, and ages (see Smith et al., 1978; Fanselow, 1990; Hartshorn et al., 1998). The operationalization of what constitutes *context* is wide and has included the odor or audio present during learning (Fagen et al., 1997; Rubin et al., 1998), the colored background on which an object was presented (Robinson and Pascalis, 2004), and the room the learner was in (Hayne et al., 2000). Further, the paradigm to test context dependency has also varied widely and has included imitation (Hayne et al., 1997), operant conditioning (Rovee-Collier and Dufault, 1991), and novel noun generalization (Goldenberg and Sandhofer, 2013). For example, in one operant conditioning paradigm, 3- and 6-month-old infants learned that their kicking behavior would cause an overhead mobile to shake, but learning was context dependent; infants' kicking behavior was lower when their crib bumper was changed to a different color and pattern than when the bumper stayed the same color and pattern as during learning (Borovsky and Rovee-Collier, 1990; Amabile and Rovee-Collier, 1991; Rovee-Collier and Dufault, 1991). Altogether, the evidence for context dependent learning is robust and suggests that children's learning is highly sensitive to contextual changes.

It is unknown if changes in the person the child interacts with will influence learning in the same way as changes in the environmental context, as shown in previous research. From one perspective, a change in the person the child interacts with should engender context dependency, for two reasons. First, people are salient and interesting to young children (Baldwin et al., 1996; Tomasello et al., 2005; Henderson et al., 2008). This early interest in and awareness of people may lead to context dependency at earlier ages. In fact, 2-year-old children's language (vocabulary use and discourse cohesion) changes as a function of who the child is interacting with (Hoff, 2010). Second, the person a child interacts with is, at its core, a perceptual stimulus (albeit far richer than, for example, any inanimate contextual stimulus). According to Tulving and Thomson (1973), context dependency is the result of a mismatch in cues between learning and testing. A change in the person a child is interacting with does cause a mismatch between perceptual cues, which should engender context dependency in the same way as other perceptual stimuli.

However, it is also possible that changes in the person the child is interacting with will not affect memory and generalization in the same way that changes in environmental context do because young children have immature source monitoring abilities (see Ackil and Zaragoza, 1995; Ruffman et al., 2001; Drummey and Newcombe, 2002). Source monitoring is the process of remembering the origin of information or knowledge (Johnson et al., 1993; Anderson, 2009). To examine developmental changes in source monitoring, researchers often ask children where they attained certain information and knowledge. Younger children have a more difficult time recalling where they learned specific information (i.e., heard it from someone, saw it themselves, or inferred it) than older children (Gopnik and Graf, 1988). Further, young children make significantly more source monitoring errors than adults if there is a delay between learning and testing or the sources are perceptually similar (Lindsay et al., 1991). The same constraints that lead children to have difficulty with source monitoring may result in less context dependent learning, particularly for younger children, when the context change involves people. That is, if children do not recall who they learned from, their memory should not be disrupted when tested by a new person.

Research suggests that development and experience likely influence the degree to which context changes affect learning (e.g., Rovee-Collier et al., 1985; Hayne et al., 1997, 2000; Hartshorn et al., 1998; Robinson and Pascalis, 2004). With experience, children are more likely to disregard irrelevant contextual information and remember relevant information, despite changes in context (Vlach and Sandhofer, 2011). It is possible that novice word learners will show more contextual dependency when the person they interact with changes between learning and testing than more experienced word learners.

The current study examines how performance on a generalization task is affected by the person the child is interacting with—what we term “person context.” Further, we examined if this effect changes across development. Three groups of children (3-, 4-, and 5-year-olds) were trained on labels for objects in a novel noun generalization task. Generalization performance was tested by asking the child to retrieve a never-before-seen category exemplar by its novel name. We were interested in whether a change in the person the child is interacting with lowers generalization scores across development. On one hand, source-monitoring research suggests that there will be no difference between context changes at a young age (i.e., 3-year-olds). On the other hand, context dependency research suggests that young children (i.e., 3-year-olds) could be most affected by person context, whereas older children could not be affected very much (i.e., 5-year-olds) Thus, we examined differences between younger and older children's generalization performance when the person the child was interacting with differed between training and testing.

## Method

### Participants

Twenty-four 3-year-olds (12 male, *M*_age_ = 41.24 months, *SD*_age_ = 2.62 months), twenty-four 4-year-olds (12 male, *M*_age_ = 54.11 months, *SD*_age_ = 2.63 months) and twenty-four 5-year-olds (9 male, *M*_age_ = 62.89 months, *SD*_age_ = 2.29 months) were randomly assigned to one of two conditions (same person context or different person context). Participants were recruited from preschools in an urban area. All children were learning English as their first language. Three additional children were not included in the analyses; one 3-year-old for failing to complete the experiment and two 4-year-olds due to experimenter error.

### Design

We manipulated whether the same experimenter conducted the training and testing phases or whether different experimenters conducted the training and testing phases. Children were first introduced to labels for eight object categories, and then were given a forced choice generalization test (e.g., Samuelson and Horst, 2007; Vlach and Sandhofer, 2011) for each of the eight categories. Pilot work in our laboratory suggested that eight was an appropriate number of categories for children of this age to comfortably complete.

### Materials

**Figure 1:**
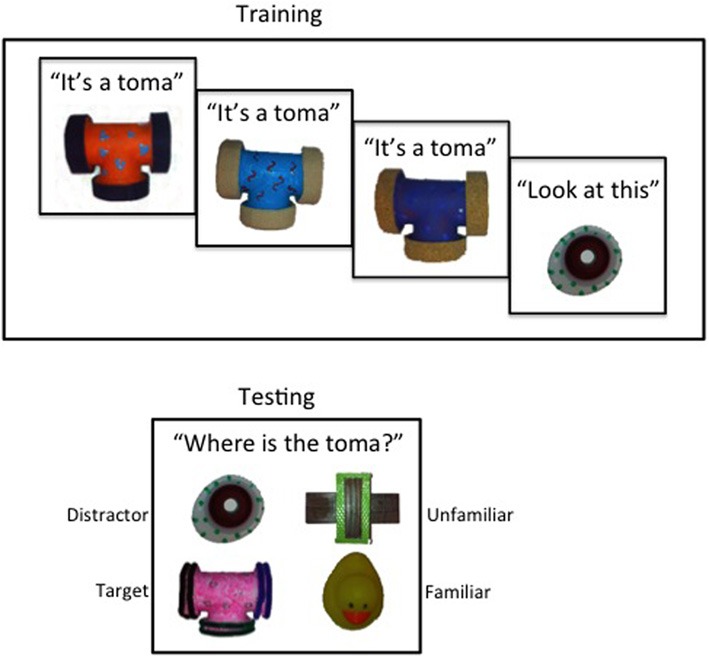
**Example of training and testing presentation**.

Novel objects were designed and constructed to be unfamiliar to the child (Figure 1). Each of the eight categories included four exemplars (three used in the training phase and one used in the testing phase). Exemplars in each category matched each other in shape, but differed in color and texture. Each object category was assigned a novel label (i.e., “wug,” “gipple,” “modi,” “dax,” “blicket,” “toma,” “fop,” and “riff”). Each category was also paired with a distractor object and an unfamiliar object, which were novel to the child, but did not share the shape of the category exemplars. The distractor object was used to ensure that a child who chose the target object during the testing phase did so in response to the request for the labeled object, and not solely based on familiarity. That is, the distractor object provided an option at test that the children had seen during training, but was not the labeled object.

Familiar objects were selected to be familiar to the child. The eight familiar objects were a duck, frog, star, pear, lion, bear, doll, and sunglasses. All objects and object label pairs were randomized and counterbalanced across participants in order to ensure that performance differences were not due to particular object shapes or labels.

### Procedure

Each child completed the training phase, followed by two filler questions and the testing phase (see Table 1). In the *same person context* condition, Experimenter 1 conducted the training phase, filler questions, and testing phase. In the *different person context* condition, Experimenter 1 conducted the training phase. After the training phase was complete, Experimenter 1 told the child that they would be leaving and another person would come to play with them. Experimenter 1 then left the room, and Experimenter 2 immediately entered and conducted the filler questions and the testing phase. The child saw neither experimenter before the training phase began. All experimenters were female and in their twenties. All experimenters' assignments to the position of Experimenter 1 and 2 were counterbalanced within and between conditions.

**Table 1:** **Example of procedure**.

**Presentation 1**	**Presentation 2**	**Presentation 3**	**Distractor**
**TRAINING PHASE**			
“It's a wug”	“It's a wug”	“It's a wug”	“Look at this”
“It's a modi”	“It's a modi”	“It's a modi”	“Look at this”
“It's a dax”	“It's a dax”	“It's a dax”	“Look at this”
“It's a toma”	“It's a toma”	“It's a toma”	“Look at this”
“It's a blicket”	“It's a blicket”	“It's a blicket”	“Look at this”
“It's a riff”	“It's a riff”	“It's a riff”	“Look at this”
“It's a gipple”	“It's a gipple”	“It's a gipple”	“Look at this”
“It's a fop”	“It's a fop”	“It's a fop”	“Look at this”
**FILLER QUESTION PHASE**			
“How old are you?”			
“When is your birthday?”			
**TESTING PHASE**			
“Where is the wug?”			
“Where is the modi?”			
“Where is the dax?”			
“Where is the toma?”			
“Where is the blicket?”			
“Where is the riff?”			
“Where is the gipple?”			
“Where is the fop?”			

#### Training phase

Each of the eight training trials included three category presentations and one distractor presentation. Experimenter 1 sat across a small table from the child and presented three successive target exemplars. Each exemplar was presented individually for 10 s and labeled with a novel word one time (e.g., “This is the toma.”). During this time, the child was allowed to touch the object. After the three target exemplars were presented, Experimenter 1 presented the distractor object for 30 s. Experimenter 1 brought attention to the distractor object without labeling it (e.g., “Look at this!”).

#### Filler questions

During this phase, the experimenter asked the child, (1) “How old are you?” and (2) “When is your birthday?” These questions were asked to create a small delay between the training and testing phases. Regardless of the child's answer, the experimenter said, “Okay.”

#### Testing phase

The testing phase was comprised of a forced choice generalization task with eight trials. The order that categories were presented in the training and testing phases were matched. In each category testing trial, the child was presented with four object choices: a target exemplar, the distractor object, a familiar object, and an unfamiliar object. These choices included items that differentiated between the correctly-labeled object (target), a familiar-but-unlabeled object (distractor), a never-before-seen-unlabeled object (unfamiliar), and an object the child already had a label for, such as a toy duck (familiar).

Once the child had a chance to touch all four object choices, the experimenter asked the child to retrieve the target object using the target name (e.g., “Where is the toma?”). When the child handed the experimenter an object, the experimenter responded neutrally (e.g., “Okay”) and began the next testing trial. A child's generalization score was calculated by summing the number of times they chose the target exemplar at test.

## Results

**Figure 2:**
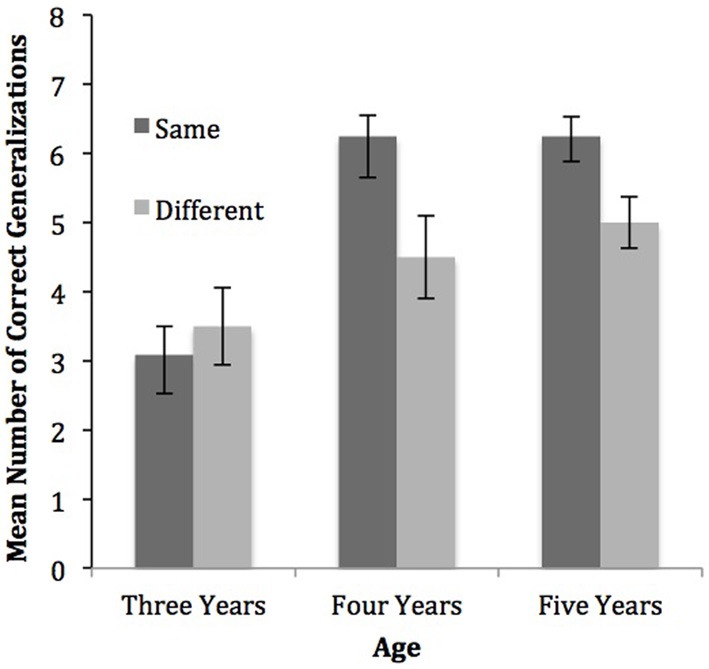
**The graph depicts the number of correct generalizations out of eight, for each age group, by the condition.** The error bars indicate standard error.

First, we asked whether the generalization scores in each condition and age group were different from chance (see Figure 2). We defined chance as 25% because there were four possible choices for children to select among during each testing trial. All group means were analyzed using a one-sample *t*-test with a comparison value of two, which is 25% of the total possible correct responses. Three-year-old children performed above chance in the same person context condition (*M* = 3.08, *SD* = 1.44), *t*_(11)_ = 2.60, *p* = 0.025, *d* = 0.75, and in the different person context condition (*M* = 3.50, *SD* = 1.93), *t*_(11)_ = 2.70, *p* = 0.021, *d* = 0.77. Similarly, 4-year-olds performed above chance in both the same person context condition (*M* = 6.25, *SD* = 1.05), *t*_(11)_ = 13.95, *p* < 0.001, *d* = 4.04, and in the different person context condition (*M* = 4.5, *SD* = 2.06), *t*_(11)_ = 4.19, *p* = 0.002 *d* = 1.21. Lastly, 5-year-olds also performed above chance in both the same person context condition (*M* = 6.25, *SD* = 0.965), *t*_(11)_ = 15.52, *p* < 0.001, *d* = 4.40, and in the different person context condition (*M* = 5.0, *SD* = 1.28), *t*_(11)_ = 8.12, *p* < 0.001, *d* = 2.34. Our question of interest was how changes in person context affected younger and older children's performance. Overall, children in every condition for each age group performed above chance levels.

Importantly, to determine if there was (1) an effect of person context condition and (2) a developmental change within this effect, we conducted a 2 (person context condition) × 3 (age) analysis of variance (ANOVA) with the number of correct generalizations as the dependent measure. The analysis revealed a main effect of person context condition, *F*_(1, 66)_ = 5.81, *p* < 0.05, *n*^2^_*p*_ = 0.081, a main effect of age, *F*_(2, 66)_ = 17.16, *p* < 0.001, *n*^2^_*p*_ = 0.342, and an interaction between person context condition and age, *F*_(2, 66)_ = 3.36, *p* < 0.05, *n*^2^_*p*_ = 0.093.

*Post-hoc* analyses were performed to determine the nature of the main effect of age. We conducted three independent samples *t*-tests, using a Bonferroni correction to maintain an alpha level of 0.05. Across conditions, 3-year-old children (*M* = 3.30, *SD* = 1.68) performed significantly lower than 4-year-old children (*M* = 5.38, *SD* = 1.84), *t*_(46)_ = −4.01, *p* < 0.001, *d* = 1.19, and significantly lower than 5-year-old children (*M* = 5.63, *SD* = 1.28), *t*_(46)_ = −5.41, *p* < 0.001, *d* = 1.56. Across conditions, 4-year-old and 5-year-old children did not perform significantly different from each other *t*_(46)_ = −0.547, *p* = 0.587, *d* = 0.15.

*Post-hoc* analyses were used to determine the nature of the interaction between person context condition and age. In order to examine the effect of person context condition at the three different age groups, we conducted three independent samples *t*-tests, using a Bonferroni correction to maintain an alpha level of 0.05. For 3-year-old children, there was no difference between generalization performance in the same person context condition and the different person context condition *t*_(22)_ = 0.53, *p* = 555, *d* = 0.25. There was a significant difference between 4-year-old children's performance in the same person context condition and the different person context condition *t*_(22)_ = 2.61 *p* = 0.016, *d* = 1.07. Similarly, 5-year-olds performed significantly higher in the same person context condition than in different person context condition *t*_(22)_ = 2.70, *p* = 0.013, *d* = 1.10.

## Discussion

In this study, we examined whether changes in the person children interacted with affected generalization in three different age groups. Four- and 5-year-old children's generalization performance was significantly higher when training and testing were conducted by the same experimenter (same person context condition) than when conducted by different experimenters (different person context condition). In contrast, 3-year-old children performed similarly in both the same and the different person context conditions. The difference between the 3-year-old and the 4- and 5-year-olds' performance is surprising because in previous contextual change studies, children consistently show an opposite pattern—one of exhibiting less context dependency with age (e.g., Hayne et al., 2000; Vlach and Sandhofer, 2011). Below we discuss two possibilities as to why the 3-year-olds were not affected by context change, although the 4- and 5-year-olds were.

One possibility as to why the 3-year-old children did not exhibit the same level of contextual dependency as seen in other studies is because their overall performance was low, and therefore, perhaps they were not learning in either condition. However, 3-year-olds performed above chance in both conditions, suggesting that they were learning in both conditions. A second possibility as to why the 3-year-olds did not exhibit context dependency is that the current study taps into the same processes that make source monitoring difficult for young children. It is possible that because 3-year-old children have more fragile source monitoring abilities than 4- and 5-year-old children, they were not disrupted by the change in person context. This explanation is in line with previous research, which suggests 3-year-old, but not 5-year-old children, have a difficult time recalling where they learned specific information (Gopnik and Graf, 1988). Because 3-year-old children show a decreased ability to monitor the source of the learned information, being tested by a new experimenter may not have disrupted their generalization. However, 4- and 5-year-olds may have more disrupted categorization because of their increased source monitoring abilities. Thus, the older children showed the classic pattern of context dependency. These developmental differences in source monitoring abilities are consistent with past research, which suggests that when two sources are similar to each other, source monitoring errors are more likely in younger children. For example, Lindsay et al. (1991) found that children were more likely than adults to make source monitoring errors when the people supplying the information were both women. However, when the people supplying the information were of different genders, children made equal amounts of source monitoring errors as adults. Thus, in the current study, 3-year-old children may have exhibited more source monitoring errors than 4- and 5-year-old children because the sources were both women, roughly the same age, and therefore, perhaps more perceptually confusable.

On the other hand, 4- and 5-year-old children did exhibit contextual dependency. That is, generalization performance was higher when the same experimenter performed training and testing, than when different experimenters performed training and testing. This suggests that the change in person context disrupted generalization as in previous research that examines context changes across a range of background contexts. In previous studies ranging across ages, tasks, and types of environmental contexts, memory is higher when the training and testing contexts match than when they do not match (Godden and Baddeley, 1975). In these studies, the mismatch in perceptual cues between training and testing disrupts memory and generalization (e.g., Rovee-Collier et al., 1985; Vlach and Sandhofer, 2011). The same disruption was likely taking place when the 4- and 5-year-olds were trained and tested by different experimenters in the current study. It is possible the 4- and 5-year-olds bound multiple aspects of the learning environment—including the source of the information—and when later asked to recall the information, the source was also included in their memory. The attention to and memory of the experimenter may have highlighted the perceptual differences between experimenters, causing context dependency.

Although we propose that these results are due to contextual dependency and source monitoring changes across development, a number of other factors could also be at play. Future research should examine the mechanisms by which children are affected by changes in person context. It is unknown what effect the level of familiarity with the experimenters had on children's generalization performance. In the current study, children in the different person context condition were tested by someone they had never seen before, and children in the same person context condition were tested by someone they had previously interacted with. Learmonth et al. (2005) found 6- to 18-month-old infants were no more likely than the control group to imitate an action when tested by a never-before-seen experimenter. Thus, the role of familiarity in the effect of person context change should be examined in future research. Further, in the current study, all experimenters were female and in their twenties. It is possible that the level of similarity between the experimenters may affect context dependency. For example, there may be fewer differences between 3- and 4-year-old children's source monitoring abilities (and more similar contextual dependency patterns) if more perceptual differences were present between experimenters (Lindsay et al., 1991). Thus, future research should examine the role of similarity in the effect of changes in person context on children's generalization.

Effects of contextual change on memory and generalization have been documented over many types of environmental contexts. However, one type of change, namely person context change, has been less examined. The current study aimed to understand whether or not children's generalization performance would be disrupted by a change in the person they were interacting with. The results suggest developmental changes in the effect of person context on categorization. These results have implications for children's classroom education, which often includes different teachers across subjects and grade levels. This study suggests that changes between teachers may affect younger and older children in different ways. The current study also has implications for studies that utilize different experimenters between training and testing to decrease experimenter bias. In the current study, all groups of children performed above chance, which is consistent with previous research showing children can transfer information learned from one experimenter to another experimenter. However, the results suggest differences in the degrees of generalization when tested by a new experimenter between different age groups. Altogether, the results of this study suggest that the person who is interacting with the child has a potent effect on children's learning.

### Conflict of interest statement

The authors declare that the research was conducted in the absence of any commercial or financial relationships that could be construed as a potential conflict of interest.
